# Therapeutic Vaccines against Human and Rat Renin in Spontaneously Hypertensive Rats

**DOI:** 10.1371/journal.pone.0066420

**Published:** 2013-06-25

**Authors:** Zhihua Qiu, Xiao Chen, Yanzhao Zhou, Jibin Lin, Dan Ding, Shijun Yang, Fen Chen, Min Wang, Feng Zhu, Xian Yu, Zihua Zhou, Yuhua Liao

**Affiliations:** Laboratory of Cardiovascular Immunology, Key Laboratory of Molecular Targeted Therapies of the Ministry of Education, Institute of Cardiology, Union Hospital, Tongji Medical College of Huazhong University of Science and Technology, Wuhan, China; School of Pharmacy, Texas Tech University HSC, United States of America

## Abstract

Vaccination provides a promising approach for treatment of hypertension and improvement in compliance. As the initiation factor of renin-angiotensin system, renin plays a critical role in hypertension. In this study, we selected six peptides (rR32, rR72, rR215, hR32, hR72, and hR215) belonging to potential epitopes of rat and human renin. The main criteria were as follows: (1) include one of renin catalytic sites or the flap sequence; (2) low/no-similarity when matched with the host proteome; (3) ideal antigenicity and hydrophilicity. The peptides were coupled to keyhole limpet hemocyanin and injected into SpragueDawley (SD) rats, spontaneously hypertensive rats (SHRs) and Wistar-Kyoto rats. The antisera titers and the binding capacity with renin were detected. The effects of the anti-peptides antibodies on plasma renin activity (PRA) and blood pressure were also determined. All peptides elicited strong antibody responses. The antisera titers ranged from 1:32,000 to 1:80,000 in SD rats on day 63. All antisera could bind to renin *in vitro*. Compared with the control antibody, the antibodies against the rR32, hR32, rR72 and hR72 peptides inhibited PRA level by up to about 50%. Complete cross-reactivity of the anti-rR32 antibody and the anti-hR32 antibody was confirmed. The epitopes rR32 and hR32 vaccines significantly decreased systolic blood pressure (SBP) of SHRs up to 15mmHg (175±2 vesus 190±3 mmHg, *P* = 0.035; 180±2 vesus 195±3 mmHg, *P* = 0.039), while no obvious effect on SD rats. Additionally, no significant immune-mediated damage was detected in the vaccinated animals. In conclusion, the antigenic peptide hR32 vaccine mimicking the ^32^Asp catalytic site of human renin may constitute a novel tool for the development of a renin vaccine.

## Introduction

Hypertension has become a leading disease in both developed and undeveloped countries [1]. Chemical drugs targeting renin-angiotensin system (RAS), including angiotensin-converting enzyme (ACE) inhibitor and angiotensin II (Ang II) receptor blocker, have exhibited excellent therapeutic effects in clinical practice. Nevertheless, the rate of controlled blood pressure (BP) is still far from satisfactory worldwide [1]. To address the compliance issue and improve therapeutic outcomes for patients, it is necessary to develop novel methods for hypertension treatment. Hypertension vaccine, where infrequent doses induce a long-term and smooth biological response, may provide benefits towards accomplishing this goal [2].

Renin vaccine interferes with the initial and rate-limiting step of RAS cascade [3]. The development of renin vaccine is seemed an attractive choice [4], [5]. Practically, renin was first used to immunize the animals as a vaccine against RAS [6]. The renin vaccine could lower the BP of animals, but was accompanied by severe autoimmune kidney diseases [7], [8]. Thus, vaccination with renin may be an effective method to block RAS and decrease BP, while the safety concerns impeded its progress. So far, no successful renin vaccines have been developed.

Renin, an aspartate protease, consists of two homologous lobes. There is a cleft between the two lobes. The active site of renin is located in the centre of the cleft [9]. The flap segment lies across the cleft, holding the substrate in the catalytic sites. The ^32^Asp and ^215^Asp residues of the active site are two main catalytic amino acids. Besides, the adjacent conserved sequences (^32/215^Asp-^33/216^Thr-^34/217^Gly-^35^Ser/^218^Thr) constitute a characteristic motif to exert catalytic activity [10]. Here, based on the catalytic sites and the flap region, six short peptides were designed. We vaccinated SpragueDawley (SD) rats and spontaneously hypertensive rats (SHRs) to evaluate the antihypertensive effect of the epitopic peptides vaccines. Wistar-Kyoto rats (WKYs) were also immunized to assess the safety of the vaccines.

## Materials and Methods

### Ethics Statement

The study was carried out in strict accordance with the Guidelines For the Care and Use of Laboratory Animals (Science and Technology Department of Huibei Province, PR China, 2005). The protocol was approved by Animal Care and Use Committee of Hubei Province (No: 00009367, 00021468). Rats were housed under specific pathogen-free conditions with 12:12 light:dark cycle at 22±2°C and 60±5% humidity. Sterile water and chow were available ad libitum. All efforts were made to minimize suffering and the procedure was performed under sodium pentobarbital anesthesia if neccessary. The procedure of obtaining human blood from patients was approved by the Ethics Committee of Tongji Medical College of Huazhong University of Science and Technology. The patient gave written informed consent and research was conformed to the guidelines of the declaration of Helsinki and its amendments.

### Peptides selection and synthesis

According to the crystal structure model of renin, we selected six B cell epitopes peptides (R32: rR32, hR32; R72: rR72, hR72; R215: rR215, hR215) as belonging to potential epitopes of rat and human renin (Table 1). The main criteria for their choice were based on: (1) the peptides include either one of renin catalytic sites or the flap sequence; (2) matched with the host proteome, the peptides have low/no-similarity; (3) the peptides have ideal antigenicity and hydrophilicity. Besides, the amino acid number of each selected peptide should be not more than 10. The peptides were synthesized by GL Ltd. (Shanghai, China) and the purity was above 98%.

**Table 1:** Sequences of the six peptides chosen as potential renin epitopes.

Name	Peptide Sequences	Location (PDB)	Species
rR32	DTGSANL	32–38	Rat
hR32	DTGSSNV	32–38	Human
rR72	TIHYGSGKVK	72–81	Rat
hR72	TLRYSTGTVS	72–81	Human
rR215	VDTGTSY	215–221	Rat
hR215	VDTGASY	215–221	Human

### Vaccines preparation and animals vaccination

The peptides were coupled to keyhole limpet hemocyanin (KLH) by sulfo-SMCC (Piecre), and then were used to subcutaneously (s.c.) immunize male SD rats (aged 6 weeks, n = 5 per group) on days 0, 14, 28, 42, and 56. The immunization dose was 500 μg. The control group rats were vaccinated with KLH only. The same volume Freund's complete adjuvant was used for the first immunization, and Freund's incomplete adjuvant for others. The peptide-specific antibody titers were screened on days 35, 49, and 63 by enzyme-linked immunosorbent assay (ELISA).

The blood pressure of animals was measured using the tail-cuff method (BP-98a, Japan). For indirect BP measurements, the animals were placed in a dark chamber at 37°C for 15 min and then transferred to a dark cage with a heating pad. The tail-cuff pressure was continuously monitored, and the signals from the pulse and pressure sensors were recorded. The BP was calculated from 20 readings for each animal. The BP measurements were performed by one person who was blinded to the identities of the groups. All the measurements were performed in a quiet environment at 20–25°C.

### Plasma renin activity (PRA), Ang II concentration mesurement and antibody preparation

On day 63, the rats were decapitated between 9 a.m and 12 a.m. Blood samples were collected and divided into two parts: one was mixed with the enzyme inhibitor mixture (1 ml blood in 50 μl inhibitor mixture including 20 μl 0.3 mol/L EDTA, 10 ul 0.32 mol/L dimercaprol and 20 μl 0.34 mol/L 8-OH-quinoline sulphate) for PRA and Ang II concentration measurement by radioimmunoassay (RIA) according to the assay kit instruction (NIBT, Beijing); the other was used for antibody experiments. The antisera were purified using protein A affinity chromatography (Bio-Rad) and additionally purified using epitope-linked gel affinity chromatography (GE Healthcare). The control antibody was purified from rats that were immunized with KLH only.

### Antibody identification and renin activity measurement

Western blotting, ELISA and RIA were used to demonstrate the specificity and cross-reactivity of the anti-peptides antibodies. The anti-peptides antibodies were used as primary antibodies. In western blotting, specific bands were detected using the chemiluminescence assay (ECL detection reagents, Pierce). In ELISA, the titers of the antisera against rat/human peptides were determined in plates which were coated with human/rat peptides of the same sites. The binding of the anti-peptides antibodies with ^125^I-renin was determined by RIA as previous description [11].

Purificated antibodies (1 µM) were preincubated with human plasma containing a high PRA (20 ng of angiotensin I/h/ml), excess renin substrate (AnaSpec) were added, then PRA was tested by RIA. Pepstatin A (Sigma) was as the positive control. In addition, different concentrations (100 nM, 1 µM, 5 µM, and 10 µM) of the anti-hR32 immunoglobulin (IgG) were also preincubated with the human plasma, then PRA was detected.

### SHRs Vaccination and BP measurement

The male SHRs aged 5 weeks were from Vital River Laboratories in China (Beijing). All animals were kept in the specific-pathogen-free room in the experimental animal center (Tongji Medical College of Huazhong University of Science and Technology, Wuhan, China). SHRs (n = 12 per group) were divided into four groups. Two groups were s.c. injected with 500 μg rR32 and hR32 vaccines adjuvanted in Freund's adjuvant respectively on weeks 6, 8, and 10. One was vaccinated with 500 μg KLH only. Another group was treated with peroral valsartan (10 mg/kg/d) (Norvatis) on week 8. The peptide-specific antibody titers were screened on weeks 9, 11, 13, 15, and 18. Half of the rats were decapitated for PRA and Ang II concentration measurement on week 12. The BP was measured using the tail-cuff method every week. The rest of animals were sacrificed on week 18. The renal cortex was taken for initial safety study.

### WKYs Vaccination and immune-complex assessment of kidney

WKYs (aged 5 weeks, Vital River Laboratories in China, Beijing) were s.c. immunized with either 500 μg rR32 or hR32 vaccine on days 0, 14, and 28. The animals were sacrificed for histological evaluation on days 7, 35, and 63. Parts of fresh renal cortex were immediately fixed in 0.25% glutaraldehyde for transmission electron microscopy (TEM). Hematoxylin and eosin (HE) staining, and Periodic Acid-Schiff (PAS) reaction staining were performed. The IgG deposition in the kidney was detected by immunofluorescence (goat anti-rat IgG antibody, FITC conjugate, Millipore). For immunohistochemical staining, the anti-rat CD14 antibody (ABBIOTEC) and anti-rat CD19 antibody (R&D system) were used.

### Statistical Analysis

Data are presented as means ± S.E. Analysis of variance (ANOVA) was used to compare variables among groups. One-way repeated measures ANOVA was used for analysis of the BP. *P*<0.05 was accepted as significant.

## Results

### Production of the anti-peptides antisera and the PRA and Ang II concentration of SD rats

**Figure 1:**
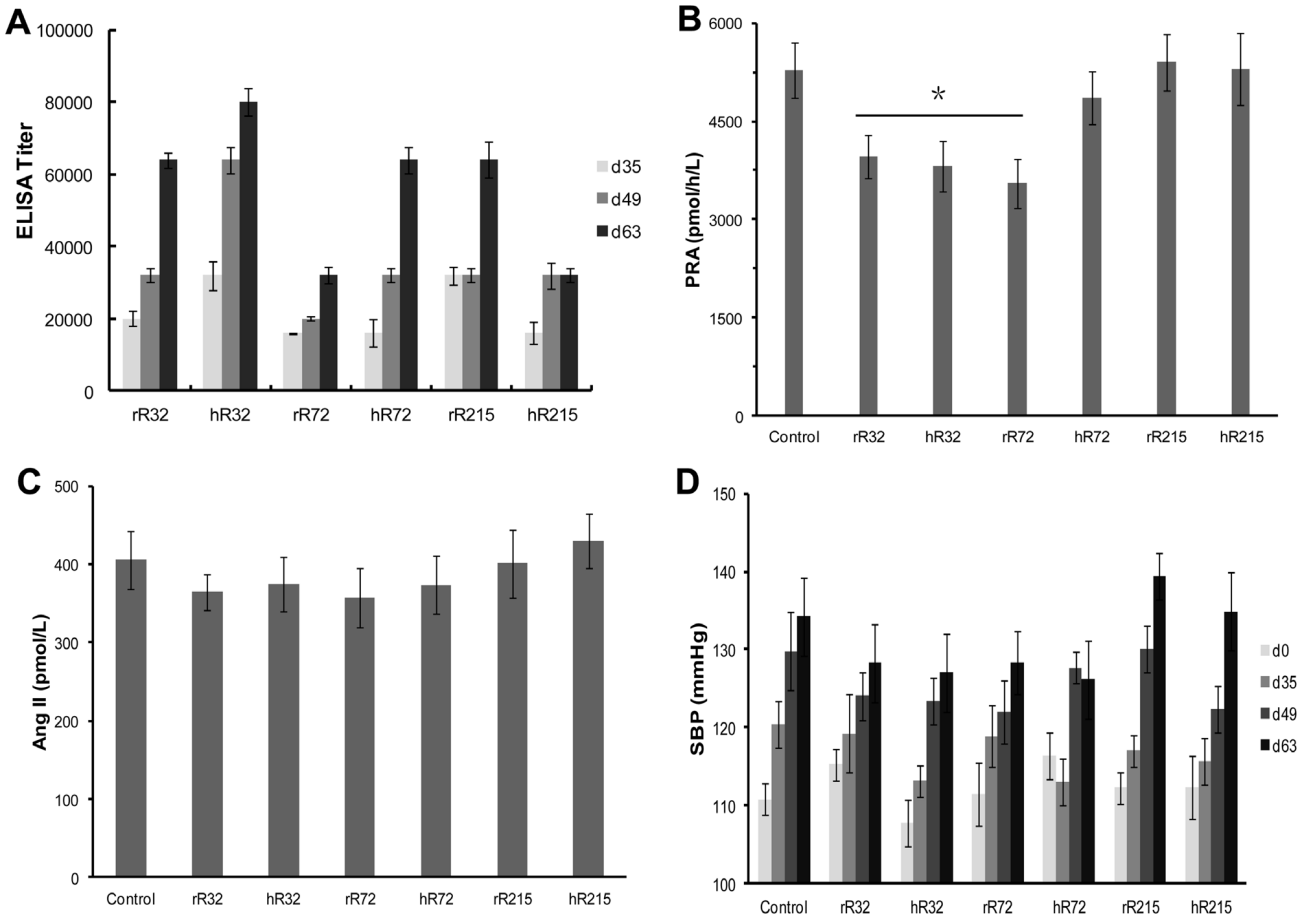
The antisera titers against peptides and the PRA and Ang II concentration of SD rats. **A.** Antibody titers were determined for antisera on days 35, 49, and 63. The six peptides antisera gave a significant A/A_0_ ratio in ELISA at dilutions ranging from 1:32,000 to 1:80,000 on day 63. **B** and **C.** On day 63, compared with the control group, the PRA was significantly decreased in the R32 and rR72 groups (**B**), while Ang II concentration only tend to be decreased in these groups (**C**). No obvious changes were observed in the hR72 and R215 groups. **D.** The BP levels had no prominent difference among groups. *P<0.05 vs the control group.

After the third, fourth and fifth injections of KLH-coupled peptides, antibody titers were determined for antisera on days 35, 49, and 63 (Figure 1A). The six peptides antisera gave a significant A/A_0_ ratio in ELISA at dilutions ranging from 1:32,000 to 1:80,000 on day 63 (Figure 1A). These indicated that the selected peptides had good antigenicity.

To evaluate RAS activity of SD rats, the PRA and Ang II concentration were determined on day 63. Compared with the control group, the PRA was significantly decreased in the rR32, hR32, and rR72 groups (Figure 1B), while Ang II concentration only tend to be decreased in these groups (Figure 1C). No obvious changes were observed in the hR72 and R215 groups (Figure 1B and 1C). Interesting, though the R32 and rR72 vaccines decreased the PRA of SD rats, they had no prominent effect on BP (Figure 1D).

### The cross-reactivity and specificity of the anti-peptides antibodies

**Figure 2:**
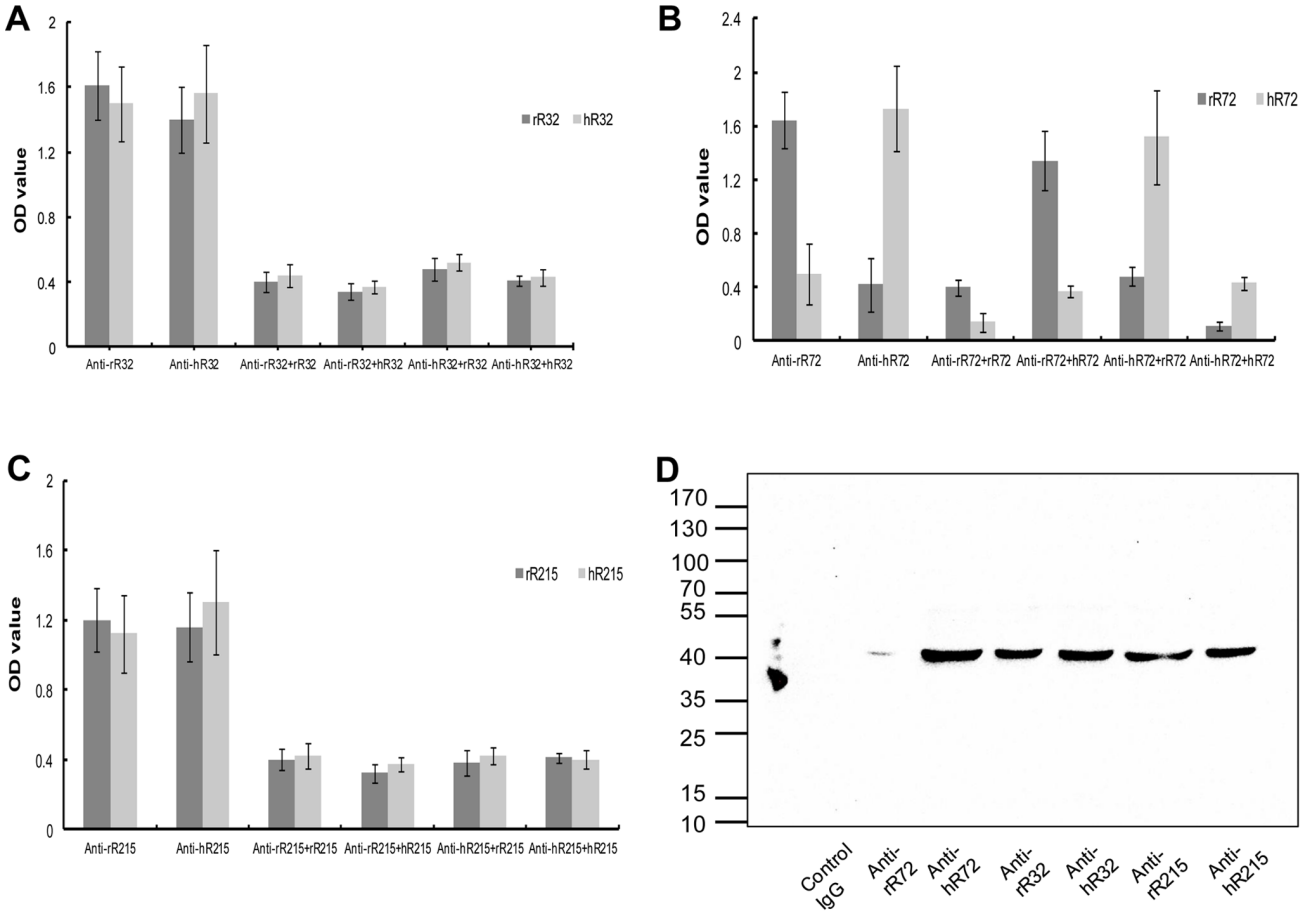
The cross-reactivity and specificity of the anti-peptides antibodies. **A–C.** The titers of the antisera against rat/human peptides were determined by an ELISA test in which plates were coated with the same sites human/rat peptides. Complete cross-reactivity of the anti-R32 and anti-R215 antibodies from rat and human sequences was confirmed, while the anti-R72 antibodies showed low cross-reactivity. **D.** The anti-peptides antibodies were used as primary antibodies, western blotting demonstrated that the anti-peptides antibodies specifically bound to renin.

To explore the possible cross-reactivity of the antibodies from the same site peptides of rat and human renin, the titers of the antisera against rat/human peptides were determined by ELISA using plates coated with the same site human/rat peptides. Complete cross-reactivity of the anti-R32 and anti-R215 antibodies from rat and human sequences was confirmed, while the anti-R72 antibodies showed low cross-reactivity (Figure 2A, 2B, and 2C).

The goal of the present study was to raise antibodies against peptide segments of the human renin molecule capable of recognizing the parent molecule and, by design, of inhibiting its activity. Western blotting demonstrated that the anti-peptides antibodies specifically bound to renin (Figure 2D). The mutual recognition of the antisera and renin was determined firstly by ELISA in which plates were coated with the recombinant human renin. The result showed that the binding of the anti-hR72 antisera with renin was the best, while the anti-rR72 was the worst (Table 2). In view of the deep position of the catalytic sites, the renin molecule may not extend its catalytic sites sufficiently in ELISA plates. The ^l25^I-labeled recombinant human renin was further used to identify the binding of the antisera with renin. We found that the antisera against the peptides, except rR72, bound to renin with a 24–51% range at a final dilution of 1:2 (Table 2). Meanwhile, when binding to renin, complete cross-reactivity of the anti-R32 and anti-R215 antibodies from rat and human sequences was confirmed, while the anti-R72 antibodies showed low cross-reactivity (Table 2).

**Table 2:** Recognition of human renin by the anti-peptides antibodies.

Peptide Antisera	ELISA A/ A_0_	^125^I-Renin Binding (%)
Control	1	2
rR32	3.36±0.12	24
hR32	3.72±0.08	28
rR72	2.41±0.15	9
hR72	5.26±0.13	51
rR215	4.24±0.14	28
hR215	4.55±0.23	35

ELISA proved that the anti-hR72 antisera showed the best binding with renin, while the anti-rR72 antisera showed the worst. In line with this finding, RIA showed the similar results.

### The purificated IgG against the R32 and R72 peptides reduced the level of PRA

**Figure 3:**
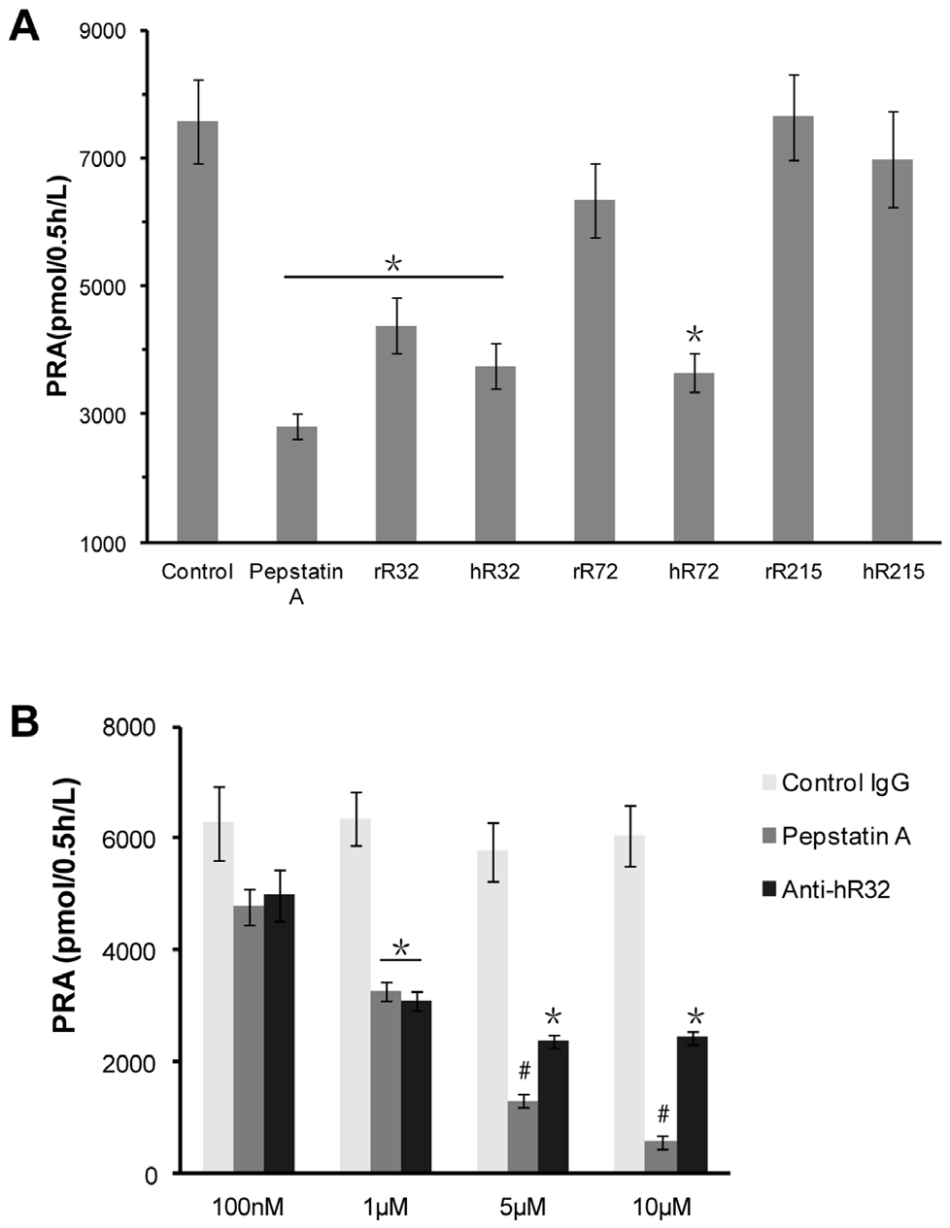
The purificated IgG against the R32 and R72 peptides reduced the level of PRA. **A.** Purificated antibodies (1 µM) were preincubated with human plasma containing a high PRA (20 ng of angiotensin I/h/ml), excess renin substrate were added, then PRA was tested by RIA. The PRA was significantly decreased by 50% in the R32 and hR72 groups, while no obvious change in the R215 groups. **B.** Different concentrations (100 nM, 1 µM, 5 µM, and 10 µM) of the anti-hR32 IgG were preincubated with the human plasma. The dose-response chart showed that more than 50% of PRA was inhibited by 5 µM anti-hR32 IgG in contrast to the control IgG. *P<0.05 and #*P*<0.01 vs the control group.

To further evaluate the effect of the antibodies on RAS activity, a high PRA plasma was used as the parent reaction pool. Additionally, to ensure zero order reaction, excess renin substrate was added. Similar to pepstatin A, the PRA was significantly decreased by 50% at 1 µM in the R32 and hR72 groups, while no change was observed in the R215 groups (Figure 3A).

Taken the low cross-reactivity into consideration, the rR72 and hR72 peptides were not suitable for animals experiment. We further dectected renin activity to value the effect of the rR32 and hR32 antibodies on PRA. We got a dose-response chart which showed that more than 50% of PRA was inhibited by 5 µM anti-hR32 IgG in contrast to the control IgG (*P* = 0.013, Figure 3B). The inhibition effect of the anti-hR32 antibody reached the plateau when the dose was 5 µM, while pepstatin A not.

### Both vaccines of rR32 and hR32 decreased BP of SHRs

**Figure 4:**
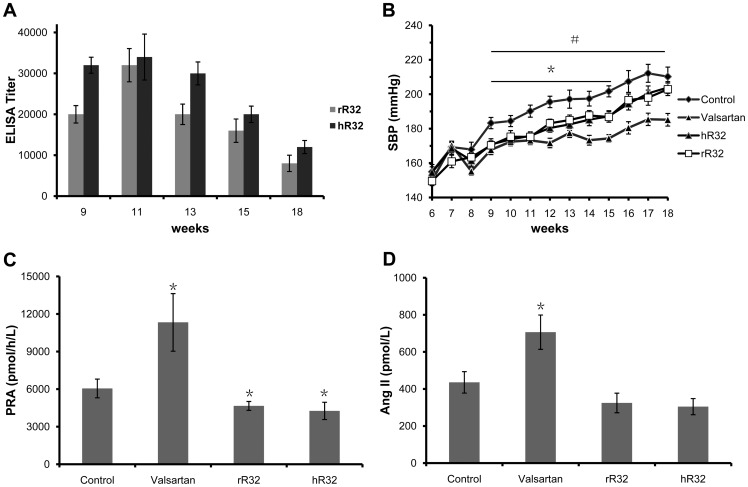
Both vaccines of rR32 and hR32 decreased SBP and PRA of SHRs. **A.** The antibody titers of the rR32 and hR32 vaccines. **B.** The rR32 and hR32 vaccines significantly decreased SBP of SHRs compared with the KLH immunization group. During the whole experiment, SBP of the valsartan group was significantly lower than that of the control group. **C.** On week 12, the PRA of the rR32 and hR32 groups was decreased compared with the control group, while the PRA of the valsartan group was increased. **D.** Ang II concentration in the vaccine groups tend to be lower than that of the control group, but no significance was observed. The Ang II concentration in the valsartan group was increased. *P<0.05 presents the vaccines groups vs the control group, and #*P*<0.01 presents the valsartan group vs the control group.

To ensure the potential anti-hypertension effect of the above peptides, the rR32 and hR32 vaccines were used to immunize SHRs for further evaluation. Oral renin inhibitor aliskiren is difficult to be obtained in China, so valsartan was selected as the positive control. ELISA screening demonstrated that the antibody titers in SHRs reached the peak on day 35 (1:40,000) and then decreased gradually (Figure 4A). Equivalent to antibody variation, the rR32 and hR32 vaccines decreased systolic blood pressure (SBP) of SHRs respectively, with a maximum decrease of 15 mmHg (175±2 vesus 190±3 mmHg, *P* = 0.035; 180±2 vesus 195±3 mmHg, *P* = 0.039), compared with the KLH group (Figure 4B). After the 16th week, although we could see the decrease tendency of SBP in the rR32 and hR32 vaccines groups compared with the control group, the statistical significance was not obvious (*P*>0.05). Meanwhile, ELISA detection indicated that the antibody titers against the rR32 and hR32 peptides declined obviously. During the whole experiment, SBP of the valsartan group was significantly lower than that of the control group. In addition, the BP in the valsartan group decreased much more steadily compared to the vaccines groups after the 12th week.

RAS activity was also detected to assess the blocking effect of the vaccines. On week 12, the PRA levels in the rR32 and hR32 vaccines groups were decreased compared with the control group (4661±355 vesus 6053±747 pmol/h/L, *P* = 0.048; 4262±690 vesus 6053±747 pmol/h/L, *P* = 0.047), while the PRA of the valsartan group was increased to 11330±2300 pmol/h/L (*P* = 0.022) (Figure 4C). Ang II concentration in the vaccine groups tend to be lower than that of the control group, but no significance was observed (324.7±52.8 vesus 435.8±57.8 pmol/L, *P* = 0.055; 304.7±43.5 vesus 435.8±57.8 pmol/L, *P* = 0.054) (Figure 4D). In the valsartan group, Ang II concentration was 706.5±92.6 pmol/L (*P* = 0.027).

### No immune-complex was observed in animals vaccinated with R32 vaccines

**Figure 5:**
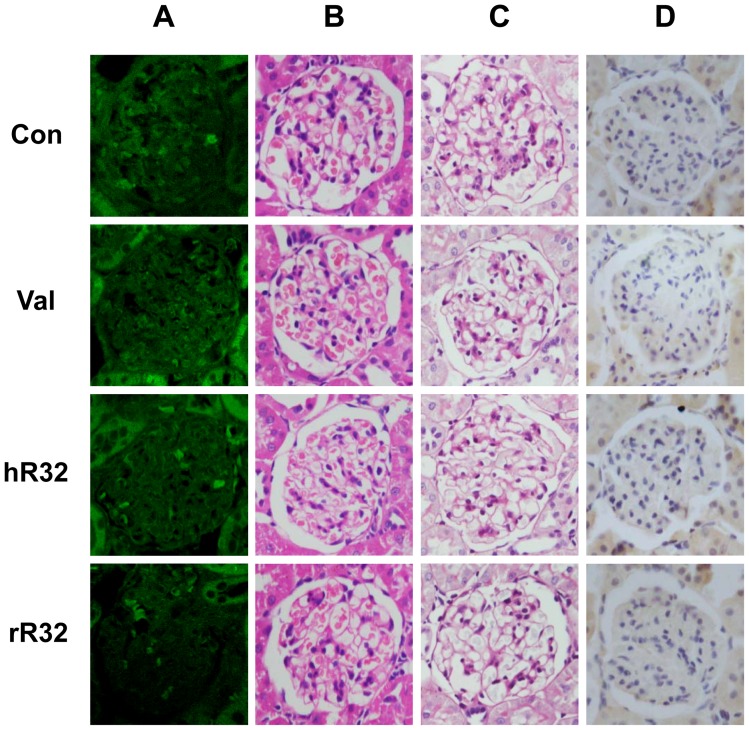
The histological safety assessment in SHRs vaccinated with R32 vaccines. **A–D.** The representative kidney images were observed under light microscopy on week 18. **A.** Compared with the control group, the IgG deposition in the kidney was not detected by immunofluorescence. Both HE (**B**) and PAS (**C**) staining showed no obvious cells proliferation and pathological changes in the mesangial region of the vaccine groups. **D.** The immunohistochemical staining against CD14 indicated no excessive activation and infiltration of macrophages in the glomeruli. Original magnification: ×400.

**Figure 6:**
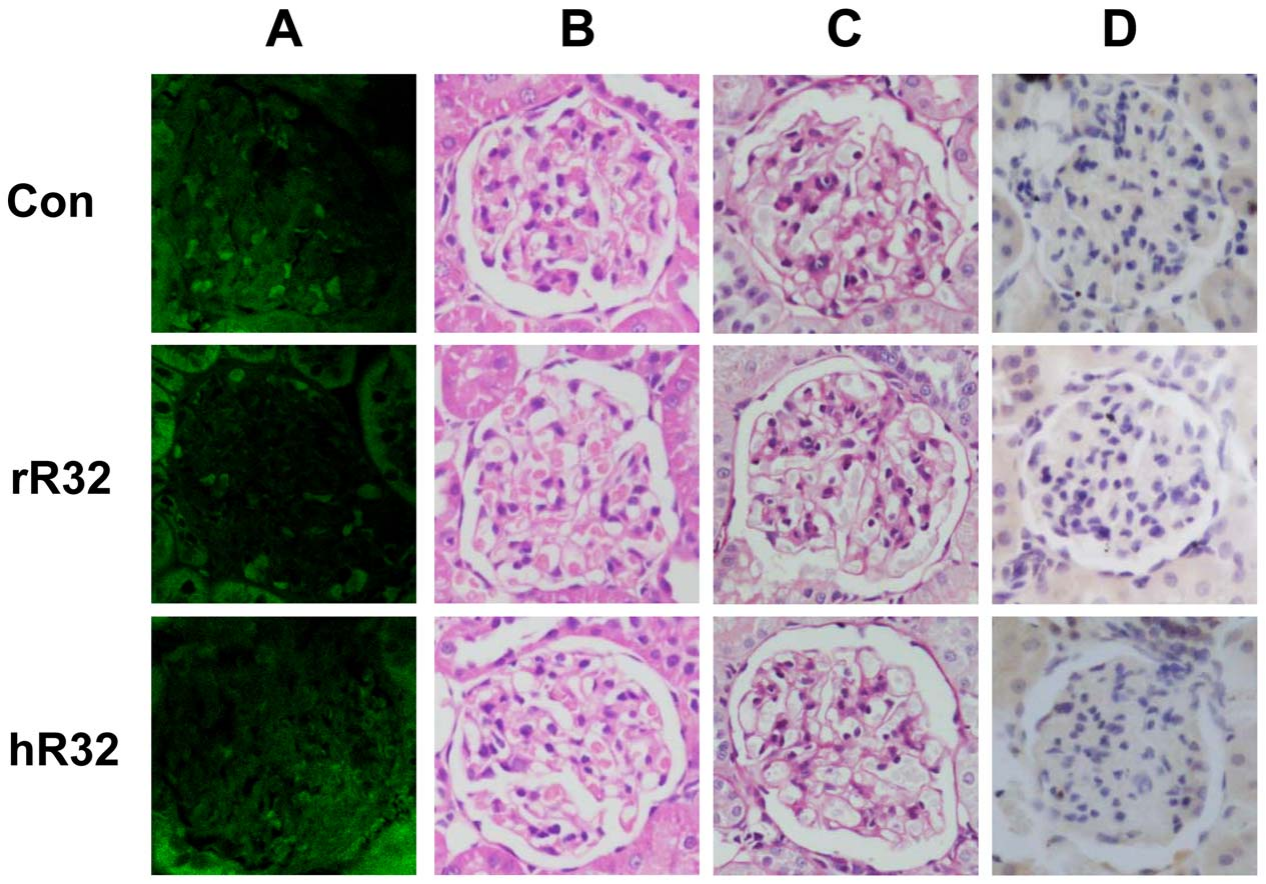
The histological safety assessment in WKYs vaccinated with R32 vaccines. **A–D.** The representative kidney images were observed under light microscopy on day 63. **A.** The IgG deposition in the kidney was not detected in the glomeruli. Both HE (**B**) and PAS (**C**) staining showed no obvious pathological changes in the mesangial region of the vaccine groups. **D.** The immunohistochemical staining against CD14 indicated no macrophages infiltration in the glomeruli. Original magnification: ×400.

**Figure 7:**
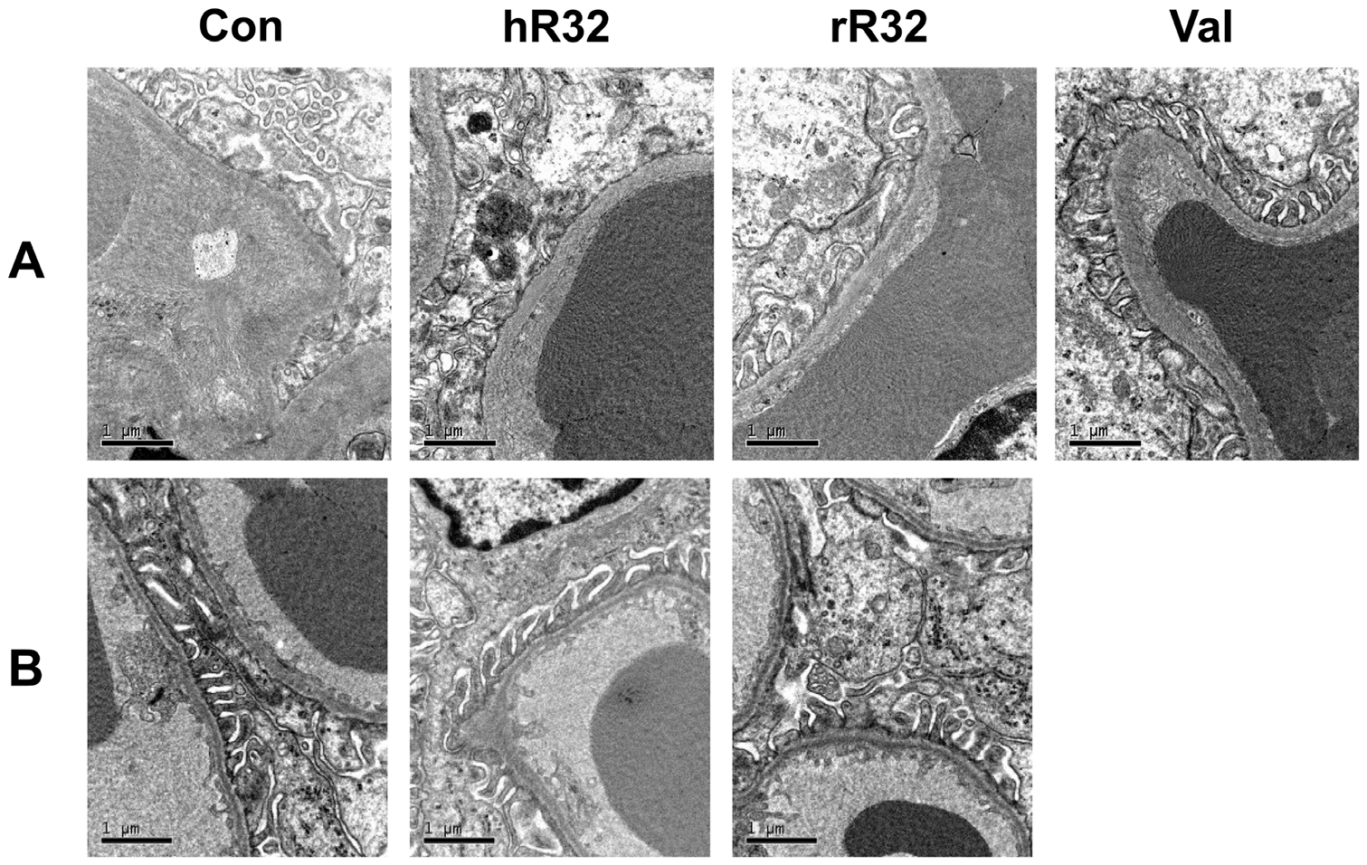
No immnue-complex was observed in the vaccinated animals under TEM. The representative kidney images showed that the basement membrane of glomeruli retained its integrity, and no characteristic immune-complex was observed in SHRs (**A)** and WKYs (**B**). Meanwhile, cells proliferation of the mesangial region was observed in the control group, but not in the vaccine goups and valsartan group (**A)**.

The histological safety assessment was performed in SHRs and WKYs. The representative kidney images were observed under light microscopy (Figure 5, 6, and S1) and TEM (Figure 7). Compared with the control group, the IgG deposition in the kidney was not detected (Figure 5A and 6A). Both HE and PAS staining showed no obvious cells proliferation and pathological changes in the mesangial region of the rR32 and hR32 vaccines groups (Figure 5B, 5C, 6B, and 6C). Moreover, the immunohistochemical staining against CD14 and CD19 indicated no excessive activation and infiltration of macrophages and B cells in the glomeruli (Figure 5D, 6D, and S1). The results of TEM showed that the basement membrane of the glomeruli retained its integrity, and no characteristic immune-complex was observed (Figure 7). Meanwhile, the cells proliferation in the mesangial region of SHRs was observed in the control group, but not in the vaccine goups and valsartan group (Figure 7A).

## Discussion

The present study demonstrated for the first time that the antisera against peptide hR32 mimicking the ^32^Asp catalytic site sequence of the human renin is capable of recognizing renin and of inhibiting part of its enzymatic activity in a dose-dependent manner. Meanwhile, the peptide hR32 vaccine could inhibit PRA and decrease SBP of SHRs.

Though the crystal structure of many protein antigens has been resolved, the definition of the antigenic determinants of a protein is not clearly established [12], [13]. Universally, the location of epitopes on the surface of the molecule, their hydrophilicity, and their segmental mobility should be evaluated. Renin is an aspartate protease that consists of two homologous lobes with the active site located in the centre of the cleft between the two lobes [9]. The flap segment lies across the cleft holding the substrate in the catalytic site. The ^32^Asp and ^215^Asp residues are the two main catalytic amino acids of human renin. And the adjacent highly conserved sequences (^32/215^Asp-^33/216^Thr-^34/217^Gly-^35^Ser/^218^Thr) comprise a motif to exert catalytic activity [10]. Based on the catalytic sites and the flap peptide, six potential epitopes were designed. Moreover, Kanduc and colleagues [14]–[16] present the low-similarity hypothesis, which defines the immune unit as a sequence with no/low similarity to the host proteome and supporting the concept that immunogenicity is preferentially associated to no/low-similarity sequences. No/low-similarity peptides means effectiveness without the risk of autoimmune phenomena. We adopted this hypothesis for peptide selection in this investigation. All our selected peptides showed excellent immunogenicity and elicited strong antibody responses.

Epitope peptide vaccine against renin was originally investigated in 1986 [17]. With the crystallographic structure of mouse renin, two epitopes (Y-211-224 and C-180-188) were identified for human renin antibodies [17]. Further research found antibodies directed against three epitopes (Y-133-144, Y-211-224 and Y-300-310) were able to inhibit renin activity [18]. Bouhnik and colleagues [11], [19] found that the renin flap region epitope was able to induce antibodies and inhibit renin enzymatic activity. However, so far, no experiments *in vivo* have been carried out and no successful renin vaccine has been developed. In our study, the antibodies against the R32 and hR72 peptides reduced the level of human PRA to less than 50%. The low cross-reactivity of the anti-R72 antibodies limited the inhibitory effect of the R72 vaccines on RAS of different species. Though RAS activity of SHRs are not higher than that of SD rats [20], [21], the peptides R32 vaccines still significantly decreased SBP of SHRs. However, the R32 vaccines had no obvious effect on SBP of SD rats. The reason may be attributed to normal PRA and regulating system of SD rats themselves. The low cross-reactivity of the anti-R72 antibodies and the lack of an appropriate animal model limited the development of the flap peptide vaccine.

Vaccination against renin with the aim of decreasing BP in hypertensive patients was firstly performed by Goldblatt [6]. Michel and colleagues [7], [8] examined the effects of active immunization against pure renin and chronic blockade of the renin substrate reaction in marmosets and rats. Renin immunization successfully led to complete blockade of RAS. Unfortunately, the effect on blood pressure against this self-antigen was accompanied by severe autoimmune disease of kidneys. Similar safety concerns were also present in the research of a vaccine against β-amyloid peptide (a 40–43 amino acid peptide) for Alzheimer's disease [22]–[24]. Therefore, the vaccination against a complete self-antigen is unlikely to be suitable, which may produce unwanted T-cell-mediated cytotoxicity against self-antigen and autoimmune diseases. The known types of immunological injuries are: (1) immune-complex deposition; (2) antibody-dependent cell-mediated cytotoxicity; and (3) activation of cytotoxic T cell against self-antigens [4], [5], [25], [26]. Immune-complex deposition is usually observed in kidney, especially in the glomerular basement membrane. In the present study, kidney damage caused by immune-complex was not detected. Immunohistochemical staining showed no inflammatory cells infiltration in the renal cortex. Nevertheless, the potential for antibody-dependent cell-mediated cytotoxicity to be caused by the vaccine will need to be further investigated. The fact that the target peptide was only 7–10 amino acids in length, shorter than the minimal T cell epitope, the number of CD8+cytotoxic T cells possibly induced and activated against the R32 peptides may be dramatically decreased [5], [25], [27]. From the results above, the R32 vaccines was seemly found to be basically safe, although further assessments are needed.

Despite the encouraging results presented here, several factors require further investigation. First, although SBP increase progressively, the RAS activity is not higher than that of normal rats in SHRs. Secondly, the low binding level of the anti-hR32 antibodies with renin did not yield substantial inhibition effect because of the deep position of the R32 peptides. Thirdly, whether the binding of renin with (pro)renin receptor was blocked by the antibody and the downstream effect are not determined [28]–[30]. Finally, emerging evidences showed the great complexity of RAS which includes ACE-Ang II-AT1 receptor axis and ACE2-Ang (1–7)-Mas receptor axis [31]. These indicate that the regulation of BP through RAS is extremely complicated. Aliskiren, a novel successful non-peptide-like renin inhibitor, has been approved for hypertension treatment [32]. However, the ALTITUDE study in the aliskiren 300 mg arm was terminated in December 2011 because of futility and an increased incidence of serious adverse events such as hyperkalemia and renal impairment [33]. Therefore, further investigation of taking renin as an intervention target is urgently indispensable.

Taken together, the hR32 vaccine mimicking the catalytic sites sequences of the human renin could inhibit human renin activity and significantly decrease SBP of SHRs. Meanwhile, evidence suggests that the vaccine was safe in the vaccinated animals. Therefore, a novel synthetic antirenin hypertension vaccine may be feasible in the future, providing a new approach to treat hypertension.

## Supporting Information

Figure S1 **No excessive activation of B cells in the vaccinated animals.** The activation of B cells in the kidneys was observed by using anti-rat CD19 antibody immunohistochemical staining. The representative kidney images were observed in SHRs (**A**) and WKYs (**B**). Compared with the control group, no excessive activation of B cells was detected in the glomeruli. Original magnification: ×400.
(TIF)
