# Supplementary figures and images for: Therapeutic Vaccines against Human and Rat Renin in Spontaneously Hypertensive Rats
Source: PLoS One. 2013 Jun 25;8(6):e66420. doi: 10.1371/journal.pone.0066420 (PMC3692469; doi:10.1371/journal.pone.0066420)

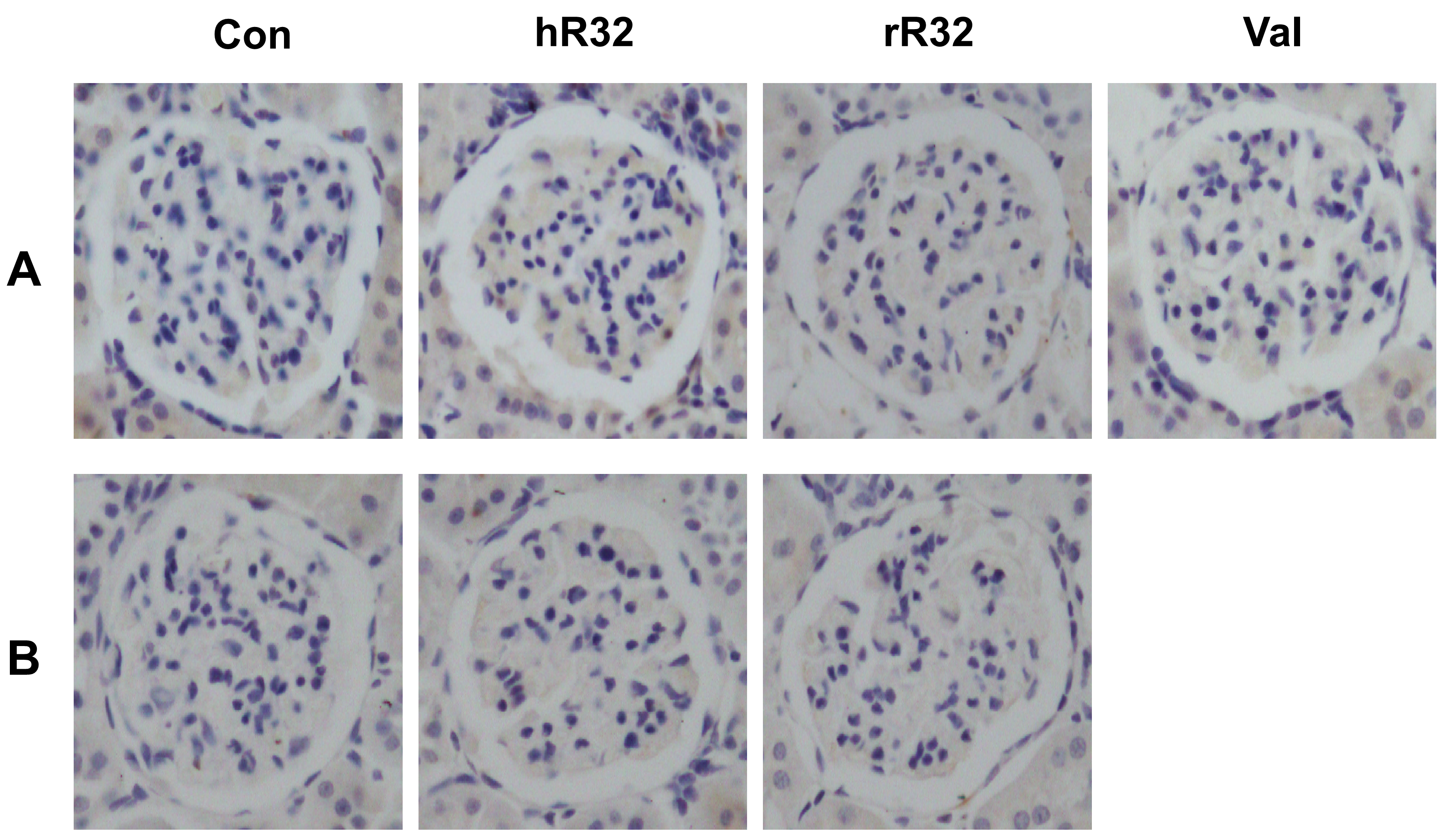

Supplement: Figure S1 — No excessive activation of B cells in the vaccinated animals. The activation of B cells in the kidneys was observed by using anti-rat CD19 antibody immunohistochemical staining. The representative kidney images were observed in SHRs (A) and WKYs (B). Compared with the control group, no excessive activation of B cells was detected in the glomeruli. Original magnification: ×400. (TIF) [file pone.0066420.s001.tif]
